# Supplementary material for: Invasive Urban Mammalian Predators: Distribution and Multi-Scale Habitat Selection
Source: Biology (Basel). 2022 Oct 19;11(10):1527. doi: 10.3390/biology11101527 (PMC9598248; doi:10.3390/biology11101527)
Supplement: Supplementary file 1 [file biology-11-01527-s001.zip › biology-1880917-supplementary.pdf]

Supplementary material for *Invasive urban mammalian predators: distribution and multi-scale habitat selection*, by Miller, K. F., Wilson, D. J., Hartley S., Innes, J. G., Fitzgerald, N. B., Miller, P., and van Heezik, Y.

Variable selection for models using a reduced set of variables

The variables rock cover, bare soil cover, and woody debris cover were removed from the parameter pool as their data distributions were strongly skewed towards 0. Mown lawn cover, low artificial cover, and building cover were removed due to strong correlation with residential garden habitat, leaving leaf litter cover as the only ground cover variable. Of the four height strata, upper canopy cover (≥12 m) was removed as it was zero at most stations.

The variable DBH was retained, as were distances from the coast (Wellington and Dunedin only), from freshwater bodies, from grassy fields/pasture, and from forest patches (specific to amenity parks and residential gardens).

Variable selection for base models

The base models for rats and mice (rodents) were created using all 17 variables, with an interaction between city and habitat type. The base hedgehog model did not have this interaction term, as preliminary results suggested it was unneeded. DBH was also omitted from the hedgehog model, as tree size was thought unlikely to affect their habitat use, owing to their limited climbing ability. Distances to coast and freshwater bodies were included in the rodent models only, because of the frequent association of Norway rats with water (Russell & Innes 2021). Because possums were almost undetected in Wellington, this city was removed from the possum model, and owing to the very low number of residential detections of possums in the other two cities, residential-only modelling was not carried out for this species.

Table S1: Mean number of days on which cats and dogs were detected on sampling transects by motion-activated cameras (two per transect) during 7-day sampling periods in three habitat types (amenity parks, forest fragments and residential gardens) in spring 2017 and autumn 2018 in Dunedin and Hamilton (n = 4 transects in each habitat) and Wellington (n = 8; n = 7 in amenity parks in autumn).

			Cats		***Dogs***	
Season	***City***	***Habitat type***	***Mean***	***SE***	***Mean***	***SE***
Spring	Dunedin	Forest	2.2	0.6	0.5	0.3
Spring	Dunedin	Amenity	5.8	0.6	0.8	0.5
Spring	Dunedin	Residential	5.2	1.2	1.5	1.2
Spring	Wellington	Forest	0.2	0.2	1.1	0.7
Spring	Wellington	Amenity	1.2	0.8	0.4	0.3
Spring	Wellington	Residential	2.5	0.5	0.2	0.2
Spring	Hamilton	Amenity	2.8	0.5	1.2	0.8
Spring	Hamilton	Forest	2.8	0.8	0	0
Spring	Hamilton	Residential	4.8	0.6	0	0
Autumn	Dunedin	Amenity	4.8	0.5	1.8	1.2
Autumn	Dunedin	Forest	2.8	0.5	0.2	0.2
Autumn	Dunedin	Residential	6.2	0.2	1.5	1.5
Autumn	Wellington	Forest	2.4	0.8	1	0.3
Autumn	Wellington	Amenity	1.3	0.4	0.9	0.5
Autumn	Wellington	Residential	4.9	0.4	1.1	0.7
Autumn	Hamilton	Amenity	1.8	0.8	1.5	0.6
Autumn	Hamilton	Forest	3.2	1.1	0	0
Autumn	Hamilton	Residential	6.8	0.2	0	0

Table S2. Summary statistics for coefficients of parameters in the residential rat model (Table 2 shows only residential-specific variables). Odds ratio (OR) is calculated at the median posterior density. Credible intervals (CI) are highest posterior density intervals (HDI) at the 90% level (5% - 95%; used because of instability at higher levels). MPE is maximum probability of effect. Statistical significance (when the 90% CI does not include 1) is denoted by + and bold text. MPE was always >0.95 (equivalent to frequentist 2-tailed p<0.1) when the 90% CI did not include 1. MPE ≥0.975 and ≥0.995 are shown as * and **, respectively and bolded. Effects of categorical variables are relative to a reference category, i.e. Season = Spring, City = Wellington, Method = Camera, level of maintenance = Low, and Compost = No.

Parameter	*OR*	*CI*	*MPE*	*Significance*
Intercept	1.88	0.25 – 13.23	0.701	
Season = Autumn	1.27	0.75 – 2.09	0.775	
City = Hamilton	1.06	0.22 - 4.65	0.527	
City = Dunedin	0.56	0.15 - 1.98	0.780	
**Method = Cards**	**0.38**	**0.18 - 0.79**	0.986	*
Distance to nearest field	2.25	0.79 - 6.96	0.897	
**Distance to forest**	**0.07**	**0.01 - 0.56**	0.987	*
Level of maintenance = Medium	0.43	0.14 - 1.16	0.918	
Level of maintenance = High	0.39	0.12 - 1.31	0.909	
**Compost = Yes**	**2.77**	**1.19 - 6.25**	0.986	*
**Proportion of native vegetation**	**0.13**	**0.02 - 0.71**	0.977	*
Garden bed cover	0.34	0.01 - 6.58	0.716	
Mown lawn cover	0.26	0.04 - 1.41	0.902	
Artificial characteristics	0.43	0.05 - 3.03	0.760	
Leaf litter	0.17	0.02 - 1.33	0.928	
**Lower canopy cover**	**25.72**	**1.35 - 540**	**0.963**	+
Shrub layer cover	0.93	0.07 - 13.2	0.516	

Table S3. Rat detection odds compared *a)* between cities, averaging over habitat types, and *b)* between cities within each habitat type, using post-hoc comparisons based on the estimated marginal means. Values are estimated odds ratios with 90% credible intervals (CI; 5–95%). MPE and significance are defined as in Table S2.

a)

City comparisons	*OR*	*CI*	*MPE*	*Significance*
**Wellington - Hamilton**	**2.84**	**1.04 - 7.97**	**0.958**	+
**Wellington - Dunedin**	**4.78**	**1.67 - 13.7**	**0.994**	*
Hamilton - Dunedin	1.69	0.54 - 4.94	0.785	

b)

City comparisons	*Habitat type*	*OR*	*CI*	*MPE*	*Significance*
**Wellington - Hamilton**	**Forest**	**5.42**	**1.24 - 26.8**	**0.962**	+
**Wellington - Dunedin**	**Forest**	**7.17**	**1.50 - 34.3**	**0.981**	*
Hamilton - Dunedin	Forest	1.33	0.19 - 7.86	0.599	
Wellington - Hamilton	Amenity	3.49	0.72 - 18.5	0.904	
**Wellington - Dunedin**	**Amenity**	**14.2**	**2.25 - 81.4**	**0.994**	*
Hamilton - Dunedin	Amenity	4.09	0.59 - 25.9	0.894	
Wellington - Hamilton	Residential	1.20	0.19 - 7.80	0.566	
Wellington - Dunedin	Residential	1.08	0.20 - 5.63	0.53	
Hamilton - Dunedin	Residential	0.90	0.15 - 6.40	0.539	

Table S4. Rat detection compared *a)* between habitat types, averaging over cities, and *b)* between habitat types within each city, using post-hoc comparisons based on the estimated marginal means. Values are estimated odds ratios with 90% credible intervals (CI; 5–95%). MPE and significance are defined as in Table S2.

a)

Habitat comparisons	*OR*	*CI*	*MPE*	*Significance*
Forest - Amenity	2.37	0.70 - 7.42	0.894	
Forest - Residential	0.52	0.13 - 1.97	0.793	
**Amenity - Residential**	**0.22**	**0.05 - 1.05**	**0.956**	+

b)

Habitat comparisons	*City*	*OR*	*CI*	*MPE*	*Significance*
Forest - Amenity	Wellington	2.18	0.60 - 7.98	0.845	
Forest - Residential	Wellington	1.59	0.44 - 6.40	0.715	
Amenity - Residential	Wellington	0.73	0.17 - 3.47	0.635	
Forest - Amenity	Hamilton	1.42	0.22 - 9.09	0.623	
Forest - Residential	Hamilton	0.36	0.04 - 3.53	0.771	
Amenity - Residential	Hamilton	0.25	0.02 - 2.67	0.829	
Forest - Amenity	Dunedin	4.37	0.58 - 38.1	0.879	
Forest - Residential	Dunedin	0.24	0.03 - 1.85	0.88	
**Amenity - Residential**	**Dunedin**	**0.05**	**0.00 - 0.57**	**0.981**	*

Table S5. Summary statistics for coefficients of parameters in the residential hedgehog model (Table 3 shows only residential-specific variables). OR, CI, MPE and significance are defined as in Table S2. Effects of categorical variables are relative to a reference category, i.e., Season = Spring, City = Wellington, Method = Camera, level of maintenance = Low, and Compost = No.

Parameter	*OR*	*CI*	*MPE*	*Significance*
Intercept	0.829	0.08 – 8.93	0.554	
**Season = Autumn**	**0.171**	**0.09 – 0.3**	**1**	**
**City = Hamilton**	**1.45**	**0.25 – 9.75**	**0.638**	
**City = Dunedin**	**24**	**4.12 - 155**	**0.997**	**
**Method = Cards**	**0.39**	**0.18 - 0.80**	**0.99**	*
Level of maintenance = Medium	1.11	0.31 - 4.17	0.555	
Level of maintenance = High	0.97	0.24 - 3.77	0.512	
Compost = Yes	0.65	0.27 – 1.69	0.77	
Proportion of native vegetation	0.3	0.04 - 1.62	0.874	
Garden bed cover	0.65	0.03 - 19.09	0.583	
Mown lawn cover	1.2	0.18 - 7.51	0.562	
Artificial characteristics	0.42	0.04 - 3.34	0.754	
Herb layer cover	2.23	0.37 - 14.09	0.773	
Lower canopy cover	4.61	0.20 - 104.06	0.792	

Table S6. Hedgehog detection compared *a)* between cities, averaging over habitat types, and *b)* between habitat types, averaging over city, using post-hoc comparisons based on the estimated marginal means. Values are estimated odds ratios with 90% credible intervals (CI; 5–95%). MPE and significance are defined as in Table S2.

A)

City comparisons	*OR*	*CI*	*MPE*	*Significance*
**Dunedin – Hamilton**	**6.55**	**2.58 – 16.6**	**0.999**	**
**Dunedin – Wellington**	**8.13**	**3.73 – 19.7**	**1**	**
Hamilton - Wellington	1.25	0.56 - 2.85	0.675	

b)

Habitat comparisons	*OR*	*CI*	*MPE*	*Significance*
**Forest - Amenity**	**0.35**	**0.14 - 0.88**	**0.969**	+
Forest - Residential	**0.54**	**0.24 - 1.21**	**0.902**	
Amenity - Residential	1.51	0.59 - 3.74	0.776	

Table S7. Summary statistics for coefficients of parameters in the residential mouse model (Table 5 shows only residential-specific variables). OR, CI, MPE and significance are defined as in Table S2. Effects of categorical variables are relative to a reference category, i.e., Season = Autumn, City = Wellington, Method = Camera, level of maintenance = Low, and Compost = No.

Parameter	*OR*	*CI*	*MPE*	*Significance*
Intercept	0.35	0.04 - 3.23	0.785	
**Season = Autumn**	**4.72**	**2.57 - 8.75**	**1**	**
**City = Hamilton**	**0.08**	**0.02 - 0.34**	**0.999**	**
**City = Dunedin**	**0.17**	**0.04 - 0.59**	**0.989**	*
Method = Cards	1.76	0.76 - 4.19	0.869	
Level of maintenance = Medium	0.85	0.29 - 2.65	0.598	
Level of maintenance = High	0.45	0.12 - 1.60	0.844	
Compost = Yes	0.98	0.46 - 2.26	0.519	
Proportion of native vegetation	1.40	0.26 - 7.24	0.634	
Garden bed cover	2.54	0.11 - 54.1	0.695	
Mown lawn cover	0.61	0.1 - 3.27	0.685	
Artificial characteristics	0.34	0.05 - 3.13	0.804	
**Distance to nearest field**	**0.17**	**0.04 - 0.65**	**0.989**	*
Leaf litter	0.8	0.09 - 6.58	0.572	
Herb layer cover	3.48	0.67 - 21.0	0.893	
Lower canopy cover	0.27	0.01 - 4.53	0.774	

Table S8. Mouse detection odds compared *a)* between cities, averaging over habitat types, and *b)* between cities within each habitat type, using post-hoc comparisons based on the estimated marginal means. Values are estimated odds ratios with 90% credible intervals (CI; 5–95%). MPE and significance are defined as in Table S2.

a)

City comparisons	*OR*	*CI*	*MPE*	*Significance*
**Wellington - Hamilton**	**3.62**	**1.77 - 7.53**	**0.999**	**
**Wellington - Dunedin**	**2.36**	**1.16 - 4.70**	**0.98**	*
Hamilton - Dunedin	0.65	0.30 - 1.41	0.823	

b)

City comparisons	*Habitat type*	*OR*	*CI*	*MPE*	*Significance*
Wellington - Hamilton	Forest	1.43	0.47 - 4.29	0.708	
Wellington - Dunedin	Forest	1.92	0.63 - 5.72	0.835	
Hamilton - Dunedin	Forest	1.33	0.36 - 4.85	0.64	
**Wellington - Hamilton**	**Amenity**	**3.21**	**1.08 - 10.2**	**0.959**	+
Wellington - Dunedin	Amenity	1.56	0.54 - 4.74	0.755	
Hamilton - Dunedin	Amenity	0.49	0.14 - 1.81	0.831	
**Wellington - Hamilton**	**Residential**	**10.2**	**2.95 - 38.3**	**0.999**	**
**Wellington - Dunedin**	**Residential**	**4.34**	**1.31 - 14.5**	**0.977**	*
Hamilton - Dunedin	Residential	0.43	0.10 - 1.73	0.841	

Table S9. Mouse detection compared *a)* between habitat types, averaging over cities, and *b)* between habitat types within each city, using post-hoc comparisons based on the estimated marginal means. Values are estimated odds ratios with 90% credible intervals (CI; 5–95%). MPE and significance are defined as in Table S2.

a)

Habitat comparisons	*OR*	*CI*	*MPE*	*Significance*
Forest - Amenity	0.91	0.45 - 1.91	0.58	
**Forest - Residential**	**4.24**	**1.92 - 9.3**	**0.999**	**
**Amenity - Residential**	**4.69**	**2.18 - 10.3**	**0.999**	**

b)

Habitat comparisons	***City***	*OR*	*CI*	*MPE*	*Significance*
Forest - Amenity	Wellington	0.74	0.29 - 1.93	0.697	
Forest - Residential	Wellington	1.69	0.66 - 4.36	0.819	
Amenity - Residential	Wellington	2.28	0.86 - 6.09	0.914	
Forest - Amenity	Hamilton	1.66	0.47 - 5.92	0.742	
**Forest - Residential**	**Hamilton**	**11.9**	**2.91 - 50.7**	**0.998**	**
**Amenity - Residential**	**Hamilton**	**7.09**	**1.74 - 30.6**	**0.989**	*
Forest - Amenity	Dunedin	0.61	0.16 - 2.05	0.743	
Forest - Residential	Dunedin	3.82	0.98 - 14.8	0.946	
**Amenity - Residential**	**Dunedin**	**6.30**	**1.64 - 24.1**	**0.988**	*

Table S10. Possum detection odds compared *a)* between cities, averaging over habitat types, and *b)* between cities within each habitat type, using post-hoc comparisons based on the estimated marginal means. Values are estimated odds ratios with 90% credible intervals (CI; 5–95%). MPE and significance are defined as in Table S2.

a)

City comparisons	*OR*	*CI*	*MPE*	*Significance*
**Dunedin - Hamilton**	**71.52**	**21.2 - 275**	**1**	**

b)

City comparisons	*Habitat type*	*OR*	*CI*	*MPE*	*Significance*
**Dunedin - Hamilton**	**Forest**	**751**	**99.2 - 6527**	**1**	**
**Dunedin - Hamilton**	**Amenity**	**8.02**	**2.22 - 37.9**	**0.994**	*
**Dunedin - Hamilton**	**Residential**	**54**	**5.06 - 668**	**0.999**	**

Table S11. Possum detection compared *a)* between habitat types, averaging over cities, and *b)* between habitat types within each city, using post-hoc comparisons based on the estimated marginal means. Values are estimated odds ratios with 90% credible intervals (CI; 5–95%). MPE and significance are defined as in Table S2.

a)

Habitat comparisons	*OR*	*CI*	*MPE*	*Significance*
**Forest - Amenity**	**42.9**	**9.7 - 186**	**1**	**
**Forest - Residential**	**3157**	**206 - 45039**	**1**	**
**Amenity - Residential**	**70.74**	**5.17 - 966**	**0.997**	**

b)

Habitat comparisons	*City*	*OR*	*CI*	*MPE*	*Significance*
**Forest - Amenity**	**Dunedin**	**412**	**45.7 - 3753**	**1**	**
**Forest - Residential**	**Dunedin**	**11494**	**752 - 201681**	**1**	**
**Amenity - Residential**	**Dunedin**	**27**	**2.78 - 285**	**0.99**	*
**Forest - Amenity**	**Hamilton**	**4.35**	**1.06 – 20.6**	**0.962**	**+**
**Forest - Residential**	**Hamilton**	**824**	**31.5 - 27184**	1	**
**Amenity - Residential**	**Hamilton**	**181**	**6.75 - 6962**	**0.995**	**

Table S12. Two city model: Rat detection compared *a)* between habitat types, averaging over cities, and *b)* between habitat types within each city, using post-hoc comparisons based on the estimated marginal means. Values are estimated odds ratios with 90% credible intervals (CI; 5–95%). MPE and significance are defined as in Table S2.

a)

Habitat comparisons	*OR*	*CI*	*MPE*	*Significance*
Forest - Amenity	0.04	0 - 5.11	0.871	
**Forest - Residential**	**2472**	**13.08 - 537992.61**	**0.994**	*
**Amenity - Residential**	**56957**	**83.59 - 58355527.63**	**0.998**	**

b)

Habitat comparisons	*City*	*OR*	*CI*	*MPE*	*Significance*
Forest - Amenity	New Plymouth	0.05	0 - 6.68	0.855	
**Forest - Residential**	**New Plymouth**	**2250**	**7.66 - 455126**	**0.99**	*
**Amenity - Residential**	**New Plymouth**	**42484**	**45.6 - 36753593**	**0.996**	**
Forest - Amenity	Tauranga	0.03	0 - 16.49	0.821	
**Forest - Residential**	**Tauranga**	**2654**	**3.63 - 2337127**	**0.98**	*
**Amenity - Residential**	**Tauranga**	**76985**	**19.19 - 543761968**	**0.991**	*

Table S13: Two city model: Hedgehog detection compared between habitat types using post-hoc comparisons based on the estimated marginal means. Values are estimated odds ratios with 90% credible intervals (CI; 5–95%). MPE and significance are defined as in Table S2.

Habitat comparisons	*OR*	*CI*	*MPE*	*Significance*
Forest - Amenity	0.13	0 - 28.1	0.738	
Forest - Residential	0.01	0 - 1.06	0.949	
Amenity - Residential	0.09	0 - 20.4	0.779	

Table S14. Two city model: Mouse detection compared *a)* between habitat types, averaging over cities, and *b)* between habitat types within each city, using post-hoc comparisons based on the estimated marginal means. Values are estimated odds ratios with 90% credible intervals (CI; 5–95%). MPE and significance are defined as in Table S2.

a)

Habitat comparisons	*OR*	*CI*	*MPE*	*Significance*
Forest - Amenity	0.04	0 - 1.87	0.939	
**Forest - Residential**	246.21	4.09 - 29764	0.991	*
**Amenity - Residential**	7331.42	54.92 - 2500470	0.999	**

b)

Habitat comparisons	*City*	*OR*	*CI*	*MPE*	*Significance*
**Forest - Amenity**	**New Plymouth**	**0.01**	**0 - 0.69**	**0.962**	**+**
Forest - Residential	New Plymouth	18.57	0.23 - 2548	0.876	
**Amenity - Residential**	**New Plymouth**	**1805**	**8.2 - 625848**	**0.994**	*
Forest - Amenity	Tauranga	0.13	0 - 17.3	0.771	
**Forest - Residential**	**Tauranga**	**3482**	**11.8 - 1101999**	**0.994**	*
**Amenity - Residential**	**Tauranga**	**29538**	**40.4 - 419899613**	**0.997**	**

Table S15. Two city model: Possum detection compared *a)* between habitat types, averaging over cities, and *b)* between habitat types within each city, using post-hoc comparisons based on the estimated marginal means. Values are estimated odds ratios with 90% credible intervals (CI; 5–95%). MPE and significance are defined as in Table S2.

a)

Habitat comparisons	*OR*	*CI*	*MPE*	*Significance*
**Forest - Amenity**	0.43	0 - 38.5	0.633	
**Forest - Residential**	303	0.72 – 176334	0.947	
**Amenity - Residential**	740	0.45 - 959342	0.941	

b)

Habitat comparisons	*City*	*OR*	*CI*	*MPE*	*Significance*
Forest - Amenity	New Plymouth	0.12	0 - 11.8	0.781	
**Forest - Residential**	New Plymouth	115.09	0.29 - 50606	0.911	
**Amenity - Residential**	New Plymouth	939	0.97 - 1647935	0.946	
Forest - Amenity	Tauranga	1.49	0 - 572	0.547	
**Forest - Residential**	Tauranga	801	0.28 - 2506084	0.93	
**Amenity - Residential**	Tauranga	558.31	0.04 - 7529472	0.874	
